# Predictors of radiotherapy non-compliance in a large public cancer center in the Philippines

**DOI:** 10.3332/ecancer.2026.2058

**Published:** 2026-01-14

**Authors:** Juzzel Ian Zerrudo, Patrick Vincent Aquino, Christian Joseph Tagal, Ma Erica Valdeabella, Christian Norwiz Buenviaje

**Affiliations:** 1 Medical Physics Section, Radiation Oncology Unit, Cancer Center, Batangas Medical Center, Batangas City, Philippines; 2 Radiation Oncology Unit, Cancer Center, Batangas Medical Center, Batangas City, Philippines; 3 Physics and Geology Unit, Department of Physical Sciences and Mathematics, College of Arts and Sciences, University of the Philippines Manila, Manila, Philippines; 4 Oncologic Drugs Administration Unit, Cancer Center, Batangas Medical Center, Batangas City, Philippines; a https://orcid.org/0009-0003-7332-7696

**Keywords:** radiotherapy, non-compliance, predictive modelling, cancer management, Philippines, LMIC

## Abstract

**Background:**

Non-compliance with radiotherapy (RT) is a critical barrier to effective cancer care, particularly in low- and middle-income countries like the Philippines. Despite a high national cancer burden, there is a lack of research on the specific factors driving RT non-compliance within the Philippine public health system. This study aimed to identify the independent predictors of non-compliance at a major public cancer center, to inform targeted interventions.

**Methods:**

This retrospective cohort study analysed the records of 448 patients with breast, cervical, head and neck, endometrial or rectal cancer who underwent curative intent RT at a large public cancer center in the Philippines between January 2022 and April 2024. Non-compliance was defined as missing two or more scheduled RT sessions. A hierarchical multivariable binary logistic regression model was used to identify independent predictors, assessing sociodemographic, clinical and seasonal/systemic factors in sequential blocks.

**Results:**

The overall non-compliance rate was 42.4%. The final multivariable model revealed that non-compliance was primarily driven by a convergence of clinical and systemic factors rather than patient demographics. The strongest predictors reflected clinical severity, specifically cancer type [cervical: odds ratio (OR) = 7.43; head and neck: OR = 3.54] and the need for a treatment replan (OR = 5.60). Systemic factors were also significant predictors, including an internal referral source (OR = 1.83) and treatment timing. Specifically, the risk of non-compliance increased for patients undergoing computed tomography simulation in the third quarter (July–September) and for those starting treatment in the fourth quarter (October–December), which are periods associated with regional climatic and socioeconomic pressures.

**Conclusion:**

In this Philippine public cancer center, RT non-compliance is driven by clinical vulnerability and dynamic systemic pressures, not static patient demographics. These findings highlight the need to shift from passive risk assessment to proactive, risk-stratified interventions. Implementing strategies such as patient navigation and support programs, adjusted for predictable seasonal pressures, can mitigate vulnerability, improve treatment adherence and ultimately enhance cancer outcomes in resource-constrained settings.

## Background

Cancer remains a significant global health burden, with nearly 20 million new cases and 10 million deaths reported in 2022 [1]. In the Philippines, the disease represents a leading cause of morbidity and mortality, accounting for approximately 190,000 new diagnoses and 113,000 deaths that same year [2, 3]. The prevalence of malignancies such as breast, lung, head and neck and cervical cancer mirrors global patterns and underscores the persistent challenges of cancer management in low- and middle-income countries (LMICs) [1, 2].

Radiotherapy (RT), the use of ionising radiation to eradicate cancer cells, is a cornerstone of modern oncology [4–7]. However, the efficacy of RT is critically dependent on strict adherence to a daily treatment schedule over several weeks. Consequently, non-compliance, defined as missing two or more scheduled sessions [8], is a major clinical challenge linked to an increased risk of recurrence, higher mortality and reduced quality of life [9–13].

Despite its clinical importance, RT compliance in LMICs like the Philippines remains understudied, a gap that is particularly concerning given the well-documented systemic barriers to care [14–17]. A recent study shows that over a third of Filipino cancer patients experience treatment delays exceeding 30 days, a problem significantly more pronounced in public hospitals [15]. These delays are rooted in significant logistical and structural challenges. The country’s archipelagic geography and its vulnerability to adverse weather force patients from at least 47 provinces to undertake long journeys, often over 100 km and including sea travel, to access care [16]. This burden is exacerbated by a severe maldistribution of resources, with 38% of all RT facilities in the country concentrated only in Metro Manila [17]. Furthermore, the entire system faces a critical shortage of specialised personnel, with only 125 radiation oncologists and 114 medical physicists for a population exceeding 110 million [17, 18].

While international research has identified patient-level predictors of RT non-compliance [8, 13, 19–21], these studies may not capture the complexities of the Philippine setting [15, 16, 18, 22, 23]. To date, no study has systematically investigated how the aforementioned systemic pressures interact with clinical factors to influence treatment adherence. This study hypothesizes that, in addition to established clinical variables, distinct contextual factors, including seasonal climatic disruptions and socio-economic pressures, are significant predictors of non-compliance. We posit that non-compliance is best understood as the outcome of interacting vulnerabilities, where a patient’s clinical burden is compounded by acute systemic and environmental pressures.

This study, therefore, aims to identify the independent predictors of RT non-compliance at Batangas Medical Center (BatMC). As the sole public RT provider for the Cavite, Laguna, Batangas, Rizal, and Quezon (Region IVA) (CALABARZON) region, a diverse area home to over 17 million people [24], BatMC offers a critical setting for this research. Findings of this study will be used for the development of an evidence-based predictive model, providing a framework for a clinical screening tool to identify high-risk patients. Ultimately, this work will enable targeted interventions to improve treatment adherence and cancer outcomes in the Philippines and other similar resource-constrained settings.

## Methods

### Study design and setting

This retrospective cohort study was conducted at the Radiation Oncology Unit of BatMC, the primary public comprehensive cancer center for the CALABARZON region. We reviewed the records of all eligible patients who initiated RT between January 2022 and had completed their treatment course by 30 April 2024, to identify independent predictors of treatment non-compliance.

### Ethical considerations

The study protocol received full ethical clearance from the Batangas Medical Center Research Ethics Committee (BatMC-RERC-2025-002). To ensure patient confidentiality, all data were fully de-identified before analysis.

### Patient eligibility

To minimise selection bias, all consecutive patients meeting the eligibility criteria during the study period were included. Inclusion criteria were: (1) a diagnosis of breast, cervical, head and neck, endometrial or rectal cancer; (2) prescription of curative intent external beam RT with conventional fractionation (1.8–2.0 Gy per fraction) and (3) completion of at least one RT session. In this study, curative intent refers to RT delivered with the primary goal of eradicating the cancer, explicitly excluding palliative regimens designed solely for symptom relief. Patients were excluded if they received palliative intent RT, were treated with a hypofractionated regimen or had medical records with substantial missing data required for the analysis.

### Definition of non-compliance

In this study, non-compliance was defined as either (1) an unplanned treatment gap of two or more consecutive scheduled sessions for patient-related reasons or (2) the unilateral discontinuation of treatment by the patient before completion of the prescribed course [8, 12]. This definition excludes treatment interruptions from institutional factors like machine maintenance or public holidays. This 2-day threshold was selected for its clinical and biological significance. Prolonging treatment allows for the accelerated repopulation of cancer cells, which can diminish locoregional tumour control by an estimated 1.2% for each day of delay in rapidly proliferating tumours like head and neck cancer (HNC) [11, 25]. This is supported by clinical evidence demonstrating that treatment breaks exceeding two days are associated with increased risks of locoregional failure and poorer disease-free survival [11].

### Data collection and variables

Data were retrospectively collected from multiple sources. Patient sociodemographic and clinical characteristics (e.g., age, sex and cancer type) were extracted from medical records. Treatment-specific parameters (e.g., RT technique, treatment dates and missed sessions) were retrieved from the ARIA^®^ oncology information system (Varian Medical Systems, Palo Alto, CA). Geographical data were determined using the 2020 Philippine Statistics Authority census to classify each patient's home barangay as urban or rural [26]. The one-way driving distance from their barangay to BatMC was calculated via the Google Maps API and served as a proxy for geographic accessibility [27]. We acknowledge that driving distance does not fully capture the complexities of travel, such as public transport availability, financial costs or the impact of local weather conditions [21]. All variables, with their operational definitions, are detailed in Table 1. For the purposes of our analysis, 'clinical severity' was conceptualised as a composite measure reflecting the cumulative physical burden on the patient, encompassing cancer type, acute treatment-related toxicities and significant clinical events like the need for a treatment replan.

### Statistical analysis

All analyses were performed using Jamovi (v2.6.4) [28]. Descriptive statistics were used to summarise patient demographics, clinical characteristics and treatment-related variables. Continuous variables were reported as means with standard deviations (SDs), while categorical variables were presented as frequencies and percentages.

Bivariate analysis was conducted to identify factors associated with non-compliance, using Pearson’s chi-square test for categorical data and independent *t*-tests (or Mann-Whitney *U* tests for non-normally distributed data) for continuous variables.

To identify independent predictors, a hierarchical binary logistic regression model was constructed. This approach was chosen to test the study's primary hypothesis that clinical and systemic factors would explain significant variance in non-compliance beyond sociodemographic characteristics. The model was built sequentially:

**Block 1 (Sociodemographic):** Age, gender, civil status, referral source, PhilHealth classification, driving distance and barangay classification.

**Block 2 (Clinical):** Cancer type, need for treatment replan, BMI and RT technique.

**Block 3 (Systemic/Seasonal):** computed tomography (CT) simulation quarter and treatment start quarter.

The final predictive model was developed using a complete-case analysis. Model fit was assessed using Nagelkerke *R*², and multicollinearity was evaluated with the Variance Inflation Factor (VIF). The model's predictive performance was measured by its classification accuracy, sensitivity, specificity and the Area Under the Receiver Operating Characteristic Curve. A two-tailed *p*-value < 0.05 was considered statistically significant for all tests.

## Results

### Patient enrollment and characteristics

From an initial 627 patients who underwent external beam RT at BatMC between January 2022 and April 2024, 448 (71.4%) were included in the final analysis. A total of 179 records were excluded due to palliative treatment intent (*n* = 94), a diagnosis other than the specified cancer types (n = 67) or incomplete data (*n* = 18). The complete patient flowchart and distribution by cancer type are detailed in Table 2.

The cohort was predominantly female (82.6%), with a mean age of 52.2 years (SD = 12.1). Most patients resided in urban barangays (54.2%) and lived within 50 km of BatMC (66.5%). The most common malignancy was breast cancer (49.8%), followed by cervical cancer (18.5%). The majority of patients were referred from within BatMC (67.9%) and received treatment using intensity-modulated radiation therapy (IMRT) (70.5%). Overall, non-compliance with RT was observed in 190 patients, corresponding to a rate of 42.4% for the cohort.

### Bivariate analysis of factors associated with non-compliance

In the bivariate analysis, several patient, clinical and systemic factors were significantly associated with treatment non-compliance (Tables 3–5). Younger age was linked to higher non-compliance, with the mean age of non-compliant patients being significantly lower than that of compliant patients (49.7 versus 54.1 years, *p* < 0.001). Similarly, non-compliant patients had a significantly lower mean BMI (23.8 versus 24.8 kg/m², *p* = 0.012).

Among categorical variables, the strongest associations were with cancer type (*X*^2^ = 68.8; *p* < 0.001) and the need for a treatment replan (*X*^2^ = 38.1; *p* < 0.001). Specifically, patients with cervical (73.5%) and head and neck (66.7%) cancer had the highest rates of non-compliance. The need for a treatment replan was a significant factor, with 86.0% of these patients being non-compliant. Referral source (*X*^2^ = 7.16; *p* = 0.007) and the seasonal timing of CT simulation (*X*^2^ = 14.3; *p* = 0.003) and treatment start (*X*^2^ = 10.2; *p* = 0.017) were also significant. In contrast, factors such as travel distance, gender, civil status and IMRT treatment type were not significantly associated with non-compliance in the initial analysis.

### Hierarchical logistic regression and model performance

To identify independent predictors, a three-step hierarchical binary logistic regression was performed. Each sequential block of predictors, sociodemographic, clinical and systemic/seasonal, contributed a statistically significant improvement to the model's predictive power (Table 6). The final model explained 32.8% of the variance in non-compliance (Nagelkerke *R*²) and demonstrated good discriminatory ability with an Area Under the ROC Curve of 0.785. At an optimised classification threshold, the model achieved an overall accuracy of 72.5% (Table 7). Collinearity diagnostics showed all VIF values were below 2.0, indicating no issues with multicollinearity.

### Independent predictors of non-compliance

After adjusting for all covariates, five factors emerged as significant independent predictors in the final multivariable model (Table 8).

Clinical factors were most prominent. Compared to patients with rectal cancer, the odds of non-compliance were over seven times higher for patients with cervical cancer odds ratio (OR = 7.43, *p* < 0.001) and over three times higher for HNC (OR = 3.54, *p* = 0.006). The need for a treatment replan was also a strong predictor, associated with over five times the odds of non-compliance (OR = 5.60, *p* < 0.001). Among systemic factors, patients referred internally within BatMC had 1.8 times the odds of non-compliance compared to those referred externally (OR = 1.83, *p* = 0.017). Finally, seasonal timing was a significant predictor. While not all individual quarters reached statistical significance against the quarter 1 (Jan–March) baseline, Likelihood Ratio Tests confirmed that the overall variables for CT Simulation Quarter (*p* < 0.001) and Treatment Start Quarter (*p* = 0.009) were significant contributors to the model (Table 9). The data revealed a clear trend of escalating risk across the year. The highest risk was associated with undergoing CT simulation in the third quarter (July–September; OR = 5.18) and starting treatment in the fourth quarter (October–December; OR = 2.03) when compared to the first quarter. Other variables, such as age, BMI and travel distance, did not remain significant predictors after adjusting for other factors.

## Discussion

This study investigated the predictors of RT non-compliance at a major public cancer center in the Philippines, identifying a rate of 42.4%. This figure is substantially higher than the rates reported in high-income settings and among other vulnerable populations, highlighting the magnified scale of this challenge in a LMIC like the Philippines [8, 21]. Our analysis revealed that non-compliance was not driven by static patient demographics but by a convergence of clinical severity with dynamic seasonal and systemic pressures. A central finding is that this convergence creates compounding vulnerability rooted in the patient's clinical condition, where the physical burden of the disease and its treatment is amplified by socioeconomic and environmental stressors.

Non-compliance in this setting appears to be an outcome of the physical burden of the disease and its corresponding treatment, a burden which is then amplified by socioeconomic and environmental stressors. The model's strongest predictors, cancer type and the need for a treatment replan, point directly to this intense physical toll. The high rates of non-compliance among patients with cervical and HNC are particularly telling, as the standard of care for these diseases is often concurrent chemo RT (CCRT) [29–34]. The addition of chemotherapy is well-documented to exacerbate acute toxicities and increase the risk of treatment interruption [35–38]. Patients with HNC, for example, endure severe mucositis, dysphagia and pain, which impair nutrition and diminish quality of life [11, 32–40]. Similarly, patients with cervical cancer face significant gastrointestinal and hematologic toxicities from pelvic irradiation, compromising their ability to tolerate daily treatment [30, 41–44]. Collectively, these severe side effects represent a major, well-documented barrier to treatment adherence [21, 36, 40, 45].

The significance of a treatment replan as a predictor underscores a critical point of patient vulnerability. While the clinical triggers for a replan may differ, ranging from a positive sign like significant tumour response to a negative one like severe weight loss [46, 47], our rationale for grouping them was to capture the disruptive event of the replan itself. In our clinical context, a replan is not a routine adjustment but a major logistical and clinical disruption, indicating that the patient’s anatomy has changed significantly, that the original treatment plan is no longer safe or optimal. This event invariably imposes new logistical burden (e.g., new CT simulation), potential financial costs and psychological stress on a patient already physically overwhelmed by daily radiation treatment [48, 49]. Given that patients with HNC and cervical cancer required the most replans, this finding reinforces the need for intensive, proactive support for these high-risk groups.

This clinical vulnerability is worsened by systemic and environmental factors. The finding that patients referred internally had higher odds of non-compliance likely reflects a combination of clinical severity and socioeconomic fragility. National data indicate that patients treated within the public hospital system face a higher risk of significant treatment delays [15]. This cohort may represent a population with greater socioeconomic constraints, often leading to diagnosis at a more advanced, symptomatic stage [50–52]. Consequently, these patients often begin RT with a greater tumour burden and poorer performance status, making them inherently more susceptible to treatment interruptions.

These pre-existing vulnerabilities are then compounded by acute seasonal shocks. The peak non-compliance risk for patients undergoing CT simulation in the third quarter (July–September) directly corresponds with two major stressors: the height of the Philippine typhoon season [53], which severely disrupts transportation [54], and the start of the academic year [55], a time of significant financial outlay for households.

This strain on family resources competes directly with the out-of-pocket expenses required for cancer care [18, 56, 57]. The heightened risk for those starting treatment in the fourth quarter (October–December) aligns with the extended Christmas season. As the country's most important cultural event, this period can divert family resources and patient focus away from the rigid demands of daily RT [58–60].

This model of interacting risks also explains why traditional sociodemographic variables like distance from treatment facility, social environment and age were not significant independent predictors. We posit that for this population, the acute and dynamic combination of severe clinical toxicity and predictable systemic shocks simply overwhelms the influence of static baseline characteristics [8, 19–21, 61–63]. In this context, the immediate, lived experience of being sick, enduring toxic treatment and facing a seasonal or financial pressure becomes a more powerful determinant of behaviour than a patient's baseline demographic profile.

### Strengths and limitations

The primary strength of this study lies in its position as the first systematic investigation into the predictors of RT non-compliance in the Philippines. By analysing a large cohort from a single public institution, we enhanced internal validity by controlling for variations in treatment protocols and produced actionable insights into non-traditional risk factors. These findings provide a crucial evidence base for health systems in the Philippines and other resource-limited settings [64]. However, they must be considered in light of several methodological limitations.

The most significant limitation is the unmeasured clinical confounding of CCRT. As CCRT is the standard of care for locally advanced cervical and HNCs that constituted our highest-risk groups, its absence from our model may have overstate the role of cancer type while understating the direct role of CCRT on treatment-induced toxicity. The addition of systemic chemotherapy is well-documented to dramatically increase the rate and severity of acute toxicities, which are primary drivers of treatment interruption and non-compliance [34–44]. Therefore, the large effect attributed to cervical cancer and HNC in our model are likely acting as strong proxies for the receipt of CCRT and its associated toxicity burden. Similarly, we also did not collect formal treatment toxicity data. The inability to control for CCRT and directly linking treatment side effects with non-adherence means that the effects attributed to these cancer types in our model likely magnified these unmeasured clinical factors. Future prospective studies are essential to disentangle the effects of cancer type from the potent, causal impact of concurrent chemotherapy on treatment compliance.

A second category of limitations relates to the use of proxies for complex socioeconomic realities. Our study lacked granular data on household income, employment details, actual travel burden and family size, necessitating the use of proxies like Philhealth membership type and driving distance. Likewise, our operational definition of ‘civil status’ grouped individuals with vastly different social support structures (e.g. a widowed patient versus young, unmarried one), which likely obscured the true relationship between a patient’s support network and their treatment compliance. The use of these proxies and their lack of statistical significance in the final model should not be interpreted as evidence that social support and access burden are unimportant, but rather that these proxies are too coarse to measure them effectively.

Another limitation relates to the study's scope. Our definition of non-compliance focused on in-treatment non-compliance and therefore excluded patients who were prescribed RT but failed to initiate treatment. This primary non-adherence, is a significant form of non-compliance that our retrospective design could not capture, as our data source only includes records for patients who have started radiation treatment. Our study’s aim was to identify predictors of interruptions during an active course of daily RT. We posit that the barriers to initiating treatment may differ substantially from those causing non-adherence once treatment has begun. The predictors for primary non-adherence represent a critical area for future research.

Furthermore, our finding that internally referred patients were less compliant is multifaceted. While we hypothesize this reflects underlying socioeconomic fragility and clinical severity at presentation, a view supported by national data showing patients in the public health system experience longer treatment delays, the referral pathway itself may introduce system-level barriers [15]. For instance, internal referrals could be associated with longer institutional wait times or more fragmented care navigation compared to externally referred patients who may arrive with a more completed initial workup. Therefore, '*Referral Source*' should be seen as a complex indicator of both patient- and system-level vulnerability that warrants further prospective investigation.

Finally, while the single-center design was a strength for internal consistency, it may limit the generalisability of some findings. Specifically, the seasonal risk patterns identified are tied to the climatic and cultural calendar of the Philippines and may not be directly applicable to other LMICs with different environmental or social patterns.

Despite these limitations, this study provides a vital foundation for understanding and addressing RT non-compliance. By highlighting the overwhelming influence of clinical severity and systemic pressures, our findings underscore the need for more integrated, tailored models of patient risk assessment.

## Conclusion

This study, conducted at a major Philippine public cancer center, concludes that RT non-compliance is driven less by static patient demographics and more by a convergence of clinical vulnerability with dynamic systemic and socio-cultural pressures. Our analysis identified patients with cervical or HNC and those requiring a treatment replan as being at significantly higher risk, reflecting the substantial burden of their disease. Crucially, this study established a powerful seasonal predictor, finding that the odds of non-compliance escalate for patients whose treatment journey begins in the third and fourth quarters of the year. This finding underscores our central thesis: that adherence is shaped not only by the severity of the disease and its treatment but also by the compounding, dynamic burden of adverse seasons and acute financial pressures.

These findings have direct implications for clinical practice and health policy. The results strongly advocate for a shift from a reactive to a proactive model of care centered on risk stratification. The predictors identified, cancer type, replan status and treatment timing, can form the basis of a simple, point-of-care screening tool to help clinicians allocate limited supportive care resources more effectively and equitably [65, 66]. This, in turn, supports the implementation of targeted patient navigation programs. A navigator could provide enhanced logistical and psychosocial support to high-risk cohorts, coordinate complex care to minimise delays and organise transport or temporary lodging, particularly during high-risk seasons.

While this study provides a critical framework, it also provides pathways for future research. To build upon this work, a prospective, multi-center, mixed-methods study designed specifically to validate our predictive model and formally evaluate the efficacy of intervention like a point-of-care screening tool and targeted patient navigation programs is warranted. A prospective design would allow for the systematic collection of key unmeasured variables, such as the use of CCRT, direct measures of household income, employment and actual travel burden thereby addressing this study's primary limitations. Furthermore, incorporating in-depth qualitative interviews would be invaluable for exploring the nuanced sociocultural factors and lived experiences behind a patient's decision to interrupt treatment, providing a deeper understanding to complement our quantitative findings.

Ultimately, these results highlight the urgent need to reframe non-compliance not as an individual failure, but as a predictable outcome of intersecting vulnerabilities. By implementing proactive, targeted interventions, such as patient navigation and seasonally-adjusted support, health systems can mitigate these identifiable risks, improve treatment adherence and optimise cancer outcomes in the Philippines and similar resource-constrained settings.

## Conflicts of interest

The authors are all employees of BatMC.

## Funding

This research received no funding.

## Author contributions

Juzzel Ian Zerrudo: Project Lead, Conceptualisation, Methodology, Data Collection, Statistical and Data Analysis, Investigation, Visualisation, Writing – Original Draft, Writing, Review & Editing, Writing of Final Manuscript, Project Administration. Patrick Vincent Aquino: Data Analysis, Visualisation, Review & Editing. Christian Joseph Tagal: Writing–Review & Editing, Analysis, Data Interpretation Ma Erica Valdeabella: Investigation, Data Collection, Writing – Review & Editing. Christian Norwiz Buenviaje: Conceptualisation, Investigation, Data Curation, Interpretation, Writing – Review & Editing.

## Figures and Tables

**Table 1.** Operational definitions and coding of study variables.

Variable	Definition/Operational Criteria
Philhealth type	This variable categorises a patient's health insurance status as a proxy for their socioeconomic standing and employment sector. Patients are classified based on their declared membership type: **Direct Contributors** (individuals with formal employment or self-employed professionals who pay premiums) or **Sponsored Members** (indigents, senior citizens, dependents and other vulnerable populations whose premiums are subsidised).
Civil status	This variable serves as limited proxy for a patient’s domestic social support structure. Patients are classified as **married** if they report being legally married or in a cohabiting/domestic partnership. Patients are classified as **single** if they report being single, widowed, divorced or separated. This dichotomisation is a methodological simplification, as each category encompasses a wide range of actual support networks.
Cancer type	This categorical variable specifies the primary malignancy diagnosed, as confirmed by histopathological analysis from a biopsy report. The cancer site is further validated as the intended target region for curative intent radiotherapy. Cancers are coded as: **Breast Ca**, **Cervical Ca**, **Rectal Ca**, **Endometrial Ca** or **HNC**. The HNC category includes malignancies of the nasopharynx, larynx and pharynx that were prescribed a curative-intent radiation dose of 60–70 Gy.
BMI	A continuous variable was calculated as weight in kilograms divided by height in meters squared. Categorisation followed WHO guidelines: • Underweight: <18.5 • Normal: 18.5–24.9 • Overweight: 25.0–29.9 • Obese: ≥30
Gender	The patient's biological sex as recorded in their official medical record, coded as male or female.
Age	A continuous variable representing the patient's chronological age in years at the start of radiotherapy. It is calculated as the interval between the patient's date of birth and the date of their first treatment session.
Driving distance	An estimate of the geographic accessibility to treatment, measured in kilometers. It represents the one-way driving distance from the patient's home barangay to BatMC, calculated using the Google Maps API.
Brgy classification	This variable describes the patient's immediate geographic and social environment using the classification from the Philippine Statistics Authority (PSA) [26]. Classifies the patient's barangay of residence as either **urban** or **rural.** This variable serves as a proxy for sociodemographic characteristics related to healthcare access. A barangay is the smallest administrative and political unit in the Philippines similar to a village or neighborhood.
CT simulation quarter	A categorical variable indicating the calendar quarter in which the patient's initial CT simulation for treatment planning was performed. The quarters are defined as: **Q1** (January–March), **Q2** (April–June), **Q3** (July–September) and **Q4** (October–December).
Treatment start quarter	A categorical variable indicating the calendar quarter in which the patient received their first fraction of radiotherapy. The quarters are defined as: **Q1** (January–March), **Q2** (April–June), **Q3** (July–September) and **Q4** (October–December).
Treatment type	This binary variable specifies the radiation therapy technique used for treatment. It is coded as **IMRT** (Intensity-Modulated Radiation Therapy) or **non-IMRT** (which includes techniques such as 3D Conformal Radiation Therapy or Conventional Radiotherapy).
Referral source	This binary variable indicates the origin of the patient's referral and serves as a proxy for their pathway into the cancer center. It is classified as **internal** for patients referred from other departments within BatMC or **external** for patients referred from other healthcare institutions. This proxy may reflect a combination of patient-level factors (e.g. socioeconomic status) and system-level factors (e.g. care navigation pathways)
Treatment replan	A binary variable indicating whether the patient required a new radiation treatment plan to be created after the initial plan was approved. A **replan** involves a new CT simulation, dose calculation and quality assurance, as prescribed by the radiation oncologist due to clinical factors such as tumour response or anatomical changes. Coded as **replan** versus **no replan**.
Missed treatments	A discrete variable quantifying patient-initiated non-adherence to the prescribed treatment schedule. It is defined as the total count of scheduled radiotherapy sessions the patient failed to attend for reasons not attributable to institutional factors (e.g., machine maintenance, official holidays).
Non-compliance	A binary variable indicating significant deviation from or failure to complete the prescribed radiotherapy schedule. A patient is classified as non-compliant if they either have a treatment gap of ≥2 scheduled sessions OR discontinue treatment entirely for patient-related reasons. This is distinct from institutional delays. Coded as **Compliant versus Non-Compliant**.

**Table 2.** Patient enrollment flowchart.

Category	Description	Number of patients (*n*)	Percentage (%)
Initial cohort	Total patients screened (January 2022–April 2024)^a^	627	100%
Exclusions	Total excluded	179	28.5% of total
	Palliative intent radiotherapy^b^	94	52.5 % of excluded
	Other cancer types^b^	67	37.4 % of excluded
	Incomplete medical records^b^	18	10.1 % of excluded
Final cohort	Total patients included in analysis	448	71.5% of total
	Breast	223	49.8% of included
	Cervical	83	18.5% of included
	Rectal	60	13.4% of included
	HNC	57	12.7% of included
	Endometrial	25	5.6% of included

a Enrollment period ended due to major LINAC maintenance

b Percentages for exclusion reasons are calculated based on the total number of excluded patients (*n* = 179)

**Table 3.** Distribution of radiotherapy compliance across cancer types.

Cancer type	Non-compliant	Compliant	Total
Cervix	61 (73.5%)	22 (26.5%)	83
HN	38 (66.7%)	19 (33.3%)	57
Endometrial	10 (40.0%)	15 (60%)	25
Breast	65 (29.1%)	158 (70.9%)	223
Rectum	16 (26.7%)	44 (73.3%)	60
Total	190 (42.4%)	258 (57.6%)	448

**Table 4.** Comparison of continuous predictors between compliant and non-compliant groups.

Predictor	Non-compliant/Mean (SD)	Compliant/Mean (SD)	Mann-Whitney *U*	*p*-value
Age (years)	49.7 (12.3)	54.1 (11.6)	19,676	<0.001
BMI (kg/m^2^)	23.8 (4.96)	24.8 (4.47)	21,115	0.012
Driving distance (km)	48.8 (70.8)	44.2 (56.3)	22,974	0.257

**Table 5.** Bivariate analysis of categorical predictors and radiotherapy non-compliance.

Predictor	Category	Total patients, *N* (% of total)	Non-compliant *N* (% category)	Compliant *N* (% category)	*X*^2^ statistic	*p*-value
Clinical factors						
Cancer type	Cervix	83 (18.5%)	61(73.5%)	22(26.5%)	68.8	<0.001
	Breast	223 (49.8%)	65 (29.1%)	158 (70.9%)		
	HN	57 (12.7%)	38 (66.7%)	19 (33.3%)		
	Endometrial	25 (5.6%)	10 (40%)	15 (60%)		
	Rectal	60 (13.4%)	16 (26.7%)	44 (73.3%)		
Treatment replan	Yes	43 (9.7%)	37 (86%)	6 (14%)	38.1	<0.001
	No	401 (90.3%)	149 (37.2%)	252 (62.8%)		
BMI category	Obese	53 (11.8%)	22 (11.6%)	31 (12%)	5.08	0.166
	Overweight	133 (29.7%)	48 (25.3%)	85 (32.9%)		
	Normal	218 (48.7%)	96 (50.5%)	122 (47.3%)		
	Underweight	44 (9.8%)	24 (12.6%)	20 (7.8%)		
Treatment type	IMRT	316 (70.5%)	132 (41.8%)	184 (58.2%)	0.179	0.672
	non-IMRT	132 (29.5%)	58 (43.9%)	74 (56.1%)		
Socioeconomic and demographic factors						
Age group	≥60 years	126 (28.1%)	45 (35.7%)	81(64.3%)	9.93	0.007
	40–59 years	254 (56.7%)	105 (41.3%)	149 (58.7%)		
	<40 years	68 (15.2%)	40 (58.8%)	28 (41.2%)		
Referral source	Internal	304 (67.9%)	142 (46.7%)	162 (53.3%)	7.16	0.007
	External	144 (32.1%)	48 (33.3%)	96 (66.7%)		
Brgy classification	Urban	243 (45.8%)	113 (46.5%)	130 (53.5%)	3.64	0.056
	Rural	205 (54.2%)	77 (37.6%)	128 (62.4%)		
Civil status	Married	308 (68.8%)	122 (39.6%)	186 (60.4%)	3.16	0.075
	Single	140 (31.3%)	68(48.6%)	72 (51.4%)		
Philhealth type	Direct contributor	232 (51.8%)	105 (45.3%)	127 (54.7%)	1.60	0.206
	Sponsored member	216 (48.2%)	85 (39.4%)	131 (60.6%)		
Gender	Female	370 (82.6%)	153 (41.4%)	217 (58.6%)	0.976	0.323
	Male	78 (17.4%)	37 (47.4%)	41 (52.6%)		
Systemic/seasonal factors						
CT simulation schedule	Q1: January–March	123 (27.5%)	40 (32.5%)	83 (67.5%)	14.3	0.003
	Q2: April–June	95 (21.2%)	42 (44.2%)	53 (55.8%)		
	Q3: July–September	100 (22.3%)	57 (57%)	43 (43%)		
	Q4: October–December	130 (29%)	51 (39.2%)	79 (60.8%)		
Treatment start schedule	Q1: January–March	134 (29.9%)	42 (31.3%)	92 (68.7%)	10.2	0.017
	Q2: April–June	103 (23.0%)	46 (44.7%)	57 (55.3%)		
	Q3: July–September	84 (18.8%)	39 (46.4%)	45 (53.6%)		
	Q4: October–December	127 (28.3%)	63 (49.6%)	64 (50.4%)		

**Table 6.** Model fit and comparison for hierarchical logistic regression.

Model step	Predictor block added	Nagelkerke *R*^2^	Change in R^2^ (Δ*R*^2^) (from previous step)	Model chi-square (*X*^2^)	df	*p*-value
1	Patient factors	0.0852	-	-	-	-
2	Clinical factors	0.2622	0.177	67.9	7	<0.001
3	Seasonal/systemic factor	0.3279	0.066	28.1	6	<0.001

Note. The Chi-Square (*χ*²), df, and *p*-value columns reflect the statistical significance of the **improvement** from the previous model. Model 1 predictors include Age, Gender, Barangay Classification, Philhealth type, Civil Status, Distance and Referral Source. Model 2 adds Cancer Type, BMI, Treatment Replan and Treatment Type. Model 3 adds the Treatment Quarter and CT Sim Quarter

**Table 7.** Predictive performance of the final model for radiotherapy non-compliance.

Performance metric	Value
Accuracy	72.5%
Sensitivity	71.6%
Specificity	73.3%
AUC	0.785

Note: Performance metrics were calculated using an optimised probability cut-off of 0.36 to classify patients as at-risk for non-compliance. AUC = Area Under the ROC Curve

**Table 8.** Logistic regression model predicting radiotherapy non-compliance.

Predictor	OR	95% Confidence Interval (CI)	*p*-value
**Clinical factors**			
**Treatment replan (Yes versus No)**	**5.6**	**2.04–15.43**	**<.001**
**Cancer type (Reference: Rectal)**			
** Cervical**	**7.43**	**2.55–21.67**	**<.001**
** Head & Neck**	**3.54**	**1.44–8.67**	**0.006**
Endometrial	2.4	0.67–8.67	0.181
Breast	1.33	0.49–3.61	0.575
Treatment type (IMRT versus Non-IMRT)	1.03	0.60–1.79	0.904
BMI (per 1 kg/m² increase)	1	0.95–1.05	0.938
**Systemic & seasonal factors**			
**CT simulation quarter (reference: Q1: Jan–Mar)** *			
Q2: April–June	1.63	0.54–4.89	0.387
** Q3: July–September**	**5.18**	**1.57–17.07**	**0.007**
Q4: October–December	0.76	0.30–1.97	0.578
**Treatment start quarter (reference: Q1: Jan–Mar)** *			
Q2: April–June	0.89	0.29–2.71	0.833
Q3: July–September	0.43	0.13–1.42	0.167
Q4: October–December	**2.03**	0.80–5.16	0.137
**Patient demographics**			
**Referral source (Internal versus external)**	**1.83**	**1.11–2.99**	**0.017**
Age (per year increase)	0.98	0.96–1.00	0.086
Gender (male versus female)	1.59	0.60–4.18	0.351
Barangay class (urban versus rural)	1.22	0.78–1.91	0.381
Civil status (partnered versus single)	0.9	0.55–1.47	0.668
PhilHealth type (direct versus sponsored)	1.03	0.63–1.68	0.898
Driving distance (per 10 km increase)	1.01	0.97–1.04	0.775

* Although not all individual quarters reached statistical significance against the Q1 reference, the overall contribution of the CT Simulation Quarter (*p* < 0.001) and Treatment Start Quarter (*p* = 0.009) variables was significant in the model based on the Likelihood Ratio Test (see Table 9)

Bold values indicate statistical significance (*p* < 0.05)

**Table 9.** Likelihood ratio tests for predictors in the final model.

Predictor	*χ* ^2^	df	*p*-value
**Clinical factors**			
**Treatment replan**	**13.51**	**1**	**<0.001**
**Cancer type**	**36.89**	**4**	**<0.001**
Treatment type	0.015	1	0.903
BMI	0.006	1	0.938
**Systemic & seasonal factors**			
**CT simulation quarter**	**17.64**	**3**	**<.001**
**Treatment start quarter**	**11.64**	**3**	**0.009**
**Patient demographics**			
**Referral source**	**5.87**	**1**	**0.015**
Age	2.9734	1	0.085
Brgy classification	0.7690	1	0.381
Philhealth type	0.0164	1	0.898
Distance	0.0819	1	0.775
Civil status	0.1832	1	0.669
Gender	0.8801	1	0.348

Bold values indicate statistical significance (*p* < 0.05)
